# Facilitating research amongst radiographers through information literacy workshops

**DOI:** 10.5195/jmla.2021.842

**Published:** 2021-01-01

**Authors:** Emily Hurt, Alison McLoughlin

**Affiliations:** 1 emily.hurt@lthtr.nhs.uk, Clinical Librarian, Lancashire Teaching Hospitals NHS Foundation Trust, Lancashire, United Kingdom; 2 asrmcloughlin1@uclan.ac.uk, National Institute for Health Research Doctoral Research Fellow, Stroke Research Team, Faculty of Health & Wellbeing, School of Nursing, University of Central Lancashire, Lancashire, United Kingdom

## Abstract

**Background:**

Despite a strong research presence in Lancashire Teaching Hospitals National Health Service (NHS) Foundation Trust (LTHTR), allied health professionals from the organization are underrepresented in developing and publicizing research that is inspired by day-to-day clinical practice and staff experiences. Two LTHTR departments, Library and Knowledge Services (LKS) and Research and Innovation (R&I), came together to enable a group of staff to develop the knowledge and skills that they needed to access information and create new “home grown” research.

**Case Presentation:**

A clinical librarian and an academic research nurse created a research engagement program in the diagnostic radiography department at LTHTR, which included the development, delivery, and evaluation of 6 workshops. Sixteen individuals took part in these workshops, and data were collected on library usage, self-efficacy in information literacy, and research output before and after their delivery. Library membership increased by 50% among diagnostic radiography staff, literature search requests from this department increased by 133%, and all participants who attended at least 1 workshop reported an increased Information Literacy Self Efficacy Scale (ILSES) score. An increase in research activity and outputs was also attributed to the program.

**Conclusions:**

This project has resulted in a set of freely available workshop plans and support resources that can be customized for other health care professionals and has won several awards for its innovative use of departmental collaboration. Through the evaluation of the program from workshop attendees and non-attenders, we have identified impacts, outputs, and barriers to engagement in order to continue to deliver this content to other departments and embed a home grown research culture at LTHTR.

## BACKGROUND

Research-engaged health care professionals are key to providing evidence-based care for patients. A review by Boaz et al. found that there “is some positive evidence…that engagement by clinicians and healthcare organizations in research can improve healthcare performance” [1]. However, a recent report from the Healthcare Improvement Studies (THIS) Institute found that staff working in the National Health Service (NHS) health care system in the United Kingdom can make valuable contributions at every stage of the research process but their potential has not yet been realized. The report identified “lack of knowledge, skills and confidence” as a major barrier to staff engaging with research [2].

NHS England has a vision of “research being everybody's business” [3] and building a culture that values and promotes research and innovation [4]. Despite a strong research presence in the Lancashire Teaching Hospitals NHS Foundation Trust (LTHTR), allied health professionals are underrepresented in developing and publicizing “home grown” research. Home grown means research developed within the organization that is inspired by day-to-day clinical practice and staff experiences at LTHTR.

Library and Knowledge Services (LKS) and Research and Innovation (R&I) are two independent departments in the trust that provide services and support for all staff and students. LKS employs thirteen staff, with five library assistants, seven librarians, and a head of the department. There are three distinct teams in LKS: Operations (staffing the enquiry desk, document delivery, marketing, and stock management); Electronic Resources (managing subscriptions, online resources, current awareness, and website development); and Clinical Librarians (literature searching, information skills training, and outreach work).

R&I is a larger team, comprising sixty-five staff across management, administrative, and clinical roles. The main remit of the department is delivering clinical research projects that are externally designed and funded. Within R&I sits the Clinical Academic Faculty (CAF), a partnership with the University of Central Lancashire (UCLan), which is the local higher education institution. The CAF is a team of three-part time employees who are responsible for increasing nurses', midwives', and allied health professionals' research and innovation capacity and capability, fostering research partnerships, and supporting the growth of locally inspired research and innovation. The CAF team are all nurses by background with an extensive range of clinical, academic, and research experience. The CAF service provides a one-stop access point for advice, support, and training for individuals and teams wanting to be involved in research, along with signposting to relevant research support networks and infrastructure. Working with LKS and external research organizations and partners, the CAF offers many options along the research pathway from support with development of research projects, applications for research internships and funding, finding and translation of evidence for practice, implementation of evidence into practice, and writing for publication support and dissemination.

LKS and the CAF are in a similar position in the wider setting of the trust, as both are nonclinical departments, occupy physical spaces away from the main hospital building, and are often trying to market their services to the same audiences. LKS at LTHTR has provided a range of training for staff and students, including skills such as literature searching and critical appraisal, but this has generally been delivered on an ad hoc basis, and the departments desired to move toward tailored training rather than a one-size-fits-all approach. We believed that if the content of a training program was shaped by the potential participants, attendees would engage with the content and acquire skills and knowledge that they identified as necessary, rather than skills and knowledge that we thought they needed. To this end, a clinical librarian and an academic research nurse representing LKS and R&I decided to create a training program that would enable LTHTR staff to develop the knowledge and skills they needed to access information and create new evidence.

In the current project, we aimed to develop, deliver, and evaluate a research engagement program for all staff in the diagnostic radiography department at LTHTR. We attempted to meet these aims by creating a set of workshop plans and support resources that are customizable to a variety of professional groups in health care settings; delivering a workshop series for diagnostic radiography staff; measuring information literacy self-efficacy, library usage, and research output before and after the workshops; evaluating the program; and identifying areas for improvement.

## CASE PRESENTATION

We selected the diagnostic radiography department for this program because of management support and a desire to develop the skill sets in the workforce. The head of the diagnostic radiography department saw our project as an opportunity to expand the skills and knowledge of staff and to raise the visibility of an often overlooked but essential department in the trust as a whole. Our program also supported the aims and objectives of the research strategy of the Society and College of Radiographers, particularly to “Expand UK radiography research capacity through development of skilled and motivated research-active members of the profession” [5]. By linking the rationale of the workshops directly to the aims of the professional body, we provided a wider and more applicable context for participants.

We invited all diagnostic radiography staff (n=141) and students (n=14), regardless of job role or level, to take part. The project team (clinical librarian and academic research nurse) ran 2 awareness-raising sessions introducing the program, the support we could provide, and the commitment required from them as individuals and a group. In addition to the awareness-raising sessions, we shared information about the program via email to all members of staff and advertising posters that were posted throughout the department. The head of the diagnostic radiography department, who oversees all staff, discussed the program with all senior team leaders and requested that they encourage attendance from their teams. Potential participants were people who had attended an awareness raising session or contacted one of the facilitators directly and registered an interest in taking part in the program (n=50).

### Development and delivery of workshops

We used SurveyMonkey to send a survey to all potential participants about their choice of workshop themes and timings. Each participant was asked to select the 6 workshops that they would most like to be delivered from a list of 16 options. Twenty-one potential participants (42%) responded to the survey, and the workshops with the most votes were:

- Overcoming Barriers to Starting Research
- What to Do with Your Research Idea
- Planning Your Research Project
- Introduction to Critical Appraisal
- An Overview of E-Journals and Databases
- Creating a Poster

The supplemental appendix provides a full list of the workshops offered in the survey.

We tailored the workshops to radiographers by using examples of research proposals, search examples, funding opportunities and bids, and conference posters from other radiographers and from LTHTR. The workshop content was developed collaboratively with both the research nurse and clinical librarian, drawing on their professional experience to decide upon the information to be covered. Each workshop contained at least one interactive element where participants were encouraged to work together, and some of the workshops required activities to be completed between sessions.

The workshops took place over a seven-month period, with sessions spaced at approximately four-week intervals. Each workshop consisted of an hour-long facilitated session delivered twice a week at different time slots to maximize attendance. After every session, a handout was distributed by email to all diagnostic radiography staff, regardless of whether they had attended, so that they could benefit from an overview of the content and links to further information. Anyone could opt out of these emails if they wished, but no one contacted us to request to be removed from the distribution list. Attendees received a continuing professional development certificate for each session.

### Measurement of engagement in research program

In total, sixteen individuals attended our workshops, and each individual attended between one and six workshops. The most well attended session was “What to Do with Your Research Idea” with eleven participants, and the least was “Planning Your Research Project” with four participants. To capture impact of the project, three measures were collected before and after the workshops: library usage, research outputs, and self-efficacy in information literacy.

### Library usage

We measured department-wide library usage and research outputs before, during, and twelve months after final workshop delivery.

At the start of the workshops, there were 16 library members with diagnostic radiography–specific job titles. Twelve months after the delivery of the workshops, there were 24 library members classified as diagnostic radiography staff, a 50% increase. There were an additional 48 new library members who were radiography students, a 200% increase (although this number could include therapeutic radiography students as well as diagnostic radiography students).

In the 12 months prior to the workshops, there were 3 requests for literature searches from diagnostic radiography staff. In the 7-month period between the delivery of the first and last workshop, there were 7 requests for literature searches (a 133% increase), and in the 12-month period after the delivery of the last workshop, there was 1 literature search request.

### Research outputs

It became evident through the program that research output was not robustly captured for home grown research at LTHTR. According to trust policies, all research should be registered with R&I, which does not always happen as many research teams identify their projects as service evaluation because of the fear of dealing with additional workload or constraints associated with registration. In reality, registering with R&I allows additional support, especially for novice researchers, which includes access to expert advice, statistical support, and widened dissemination opportunities.

As a result, there was no audit of home grown research activity in the diagnostic radiography department prior to the program to allow change over time to be documented. Research outputs such as presentations at conferences or posters were not centrally recorded in the trust, but work is underway to develop a database where future activity will be captured. Research activity after the program was informally captured from participant evaluations.

### Self-efficacy in information literacy

We utilized the Information Literacy Self Efficacy Scale (ILSES) [6] before and after the series of workshops to measure participants' self-efficacy in relation to information literacy. Participants registered with a nickname to maintain anonymity and elicit authentic answers. They rated 28 statements on a scale of 1 to 7, where 1 was almost never true and 7 was almost always true; for example, “I feel confident and competent to decide where and how to find the information I need” [6]. Higher scores indicated higher levels of self-efficacy for information literacy.

Twenty-three diagnostic radiography staff members completed the initial ILSES. Fourteen of those then went on to attend at least 1 of the 6 workshops, and 10 completed the final ILSES, which measured information literacy self-efficacy after all of the workshops had been delivered. Given the small number of participants (n=10), we were unable to complete statistical analyses beyond descriptive measures. Initial ILSES scores ranged from 62 to 151, with a mean of 107, and final ILSES scores ranged from 131 to 168, with a mean of 152. All participants who attended at least 1 workshop had an increased ILSES score, with score increases ranging from 10 to 89 and a mean score increase of 44. The largest increases in scores were for using the library catalogue, locating useful information sources in the library, and evaluating web sources.

### Evaluation of the program

The program was designed with built-in evaluation to allow the authors to examine the work and identify areas for improvement. There were two areas of evaluation: individual workshops and non-attenders. Evaluation was mainly formative, looking at process and implementation, and we evaluated impact for participants.

#### Individual workshops.

Three weeks after completion of each workshop, all participants (n=16) were given the opportunity to evaluate the session through five questions:

- Would you recommend this workshop to others?
- What did you like about the workshop?
- What did you dislike about the workshop?
- Did you take any action as a result of attending the workshop? If yes, what did you do?
- Is there anything else you would like to have covered in this session? If yes, please tell us what.

We sent evaluations to attendees of each workshop; because some of the sixteen individual attendees attended multiple workshops; there were forty-two total evaluations sent. Of the possible forty-two evaluations, sixteen were received. One hundred percent of respondents said they would recommend the workshop to others. Selected responses are provided in Table 1.

#### Non-attenders.

We requested feedback via an anonymous questionnaire given to all staff in the diagnostic radiography department via email and via face-to-face feedback sessions. All respondents completed questionnaires during face-to-face sessions. The aim of collecting feedback was to identify factors that prevented staff members from attending the program. The authors felt strongly that it was important to identify areas in which participation could be improved with future cohorts. We posed three questions to non-attenders:

- Do you think that information literacy and research skills are important to your role? And why/why not?
- What prevented you from attending the workshop sessions? (Be as honest and specific as you like.)
- What would you like to see from Research and Innovation and Library Services to support you and/or the department in the future?

Out of 139 non-attenders in the diagnostic radiography department, 16 people (12%) attended the face-to-face feedback sessions, and 11 questionnaires were completed. Eighty-one percent (n=9) of respondents thought that information literacy and research skills were important to their role, and 18% (n=2) did not think that information literacy and research skills were important to their role. One stated, “not for my role as there is no progression opportunities. However for radiography overall I think it plays an important part.” Seventy-three percent of respondents (n=8) felt information literacy was important in terms of improving clinical practice and developing diagnostic radiographers as a profession. Table 2 provides selected responses from these sessions.

## CONCLUSIONS

### Project impacts and outputs

Our project was innovative in that it tackled some of the barriers to delivering information literacy training in a pressurized work environment. The project has resulted in a set of workshop plans and support resources that could be used by other information professionals in a health setting [7], and these were launched at LILAC 2017 [8].

The project has been showcased at several national LKS and R&I conferences and at UK Radiological and Radiation Oncology Congress (UKRCO) as a poster presentation. Internally, the project won “Partnership of the Year” at a trust awards event. It was also voted the Gold Library and Information Health Network North West (LIHNN) Quality Improvement Award.

**Table 1:** Selected responses from individual workshop evaluations

Question	Selected comments
What did you like about the workshop?	“Practical session which enabled you to carry out exercise to consolidate information which is given out.” “Informative Fun Made me realise I am not the only person who has felt ‘underqualified' for tasks before.” “A chance to get away from the workplace and think about a different subject. Relaxed informative atmosphere. Appreciated amount of prep work done by the presenters to make it interesting.”
What did you dislike about the workshop?	“Nothing.” “Over lunchtime.” “Not many attendees so discussion was more limited than it could have been—but still good.”
Did you take any action as a result of attending the workshop? If yes, what did you do?	“Went back onto the intranet and tried using the different sources.” “Not really, however this was due to lack of time and not inclination. Hopefully will be able to progress in the new year.” “Yes, I have spoken to [librarian] about PhD routes, as well as attending a research event at [University of Central Lancashire] UCLan. I am also arranging to see [librarian] for a literature search.”
Is there anything else you would have liked to have covered in this session? If yes, please tell us what.	“No very thorough” “No—very comprehensive”

**Table 2:** Selected responses from non-attender feedback sessions

Question	Selected comments
What prevented you from attending the workshop sessions? (Be as honest and specific as you like.)	“Do not have enough staff to let people attend, during lunch people want to be away from the department.” “I am new to the hospital so was not aware of them.” “I found it hard to understand and get my head around the topic in general.”
What would you like to see from Research and Innovation and Library Services to support you and/or the department in the future?	“Online learning/videos to be able to ‘dip into' when time is available and perhaps a person to contact if there was queries or assistance needed.” “Individual meetings to discuss ideas and ways forward.” “Listening to the talks and reading the informations *[sic]* the research and innovations and library services offer all the support I would need.” “Assistance in locating and accessing resources specific to best practice in radiography.”

The collaboration between LKS and R&I led to several positive impacts within and beyond our project. Partnership working in this context was considered the best approach, as this not only strengthened existing relationships between the two departments and individual staff, but it also meant that diagnostic radiography staff and students saw the two departments as connected to each other, demonstrating the expertise that both could contribute to the research journey. This collaboration has been mutually beneficial to all departments involved and has direct implications for patient care, professional development of staff, and embedding of a research culture. LKS and R&I are keen for the program to continue with a different professional group or department. Although external funding secured this project initially, the facilitators are now supported to adapt the existing content to deliver to another professional group at the trust as part of their existing job roles. A short video has been created to advertise the benefits to other potential future cohorts [9].

### Participant impacts and outputs

In the workshop evaluations, participants reported positive experiences with the program as a whole. They attributed the following tangible outcomes to attendance:

- One participant successfully submitted a poster to UKRCO.
- One participant was accepted to the trusts' new Clinical Academic Trainees program and is released from her role one day a week to carry out an evidence-based service improvement project.
- Two participants created a poster to share the results of a patient satisfaction survey.

Following the findings that home grown research activity was poorly captured and audited by R&I, work has been ongoing to improve these processes. There is an awareness that research activity from allied health professionals in particular goes unnoticed, and a conscious effort is being made by both allied health professional leads and R&I to rectify this.

Although there has not been an overall increase in the number of literature searches conducted by the clinical librarian for this professional group since the completion of the workshops, there has been an increase in communication between diagnostic radiography staff and LKS in general. The clinical librarian was asked to speak at a regional radiography study day, to provide an example of how the trust supports staff with continuing professional development and encourages research activity.

Our project had some limitations. It could well be that the participants increased their ILSES scores because they wanted the researchers to be able to demonstrate that their project had been successful. The researchers had developed camaraderie with the participants as a result of meeting with them on a regular basis over a period of months, and it may be that the participants wanted the researchers to succeed, as described in the Hawthorne effect [10].

The final number of workshop participants was small, representing only 32% of potential participants (those who had registered an interest in taking part) and 10% percent of the diagnostic radiography department as a whole. However, the researchers were fully aware that this project was not going to attract high numbers of participants, and we felt it was more important that those who attended were people who wanted to engage with the program and its objectives. Two members of staff confided in a senior manager that completing the ILSES had dissuaded them from taking part in the program because they were concerned that the content would not be at the right level for them. We were unaware of this until the program and evaluations had been completed; therefore, we were unable to gauge the effect of ILSES completion on attendance.

The program has since been delivered to 28 therapeutic radiography staff in the trust, reaching 30% of the overall department. We find these numbers encouraging, especially in comparison to our diagnostic radiography program that reached 10% of the staff. Moving forward, there are plans to deliver the program to other groups, ensuring that senior management buy-in is present in order to enable maximum engagement and participation. As a result of presentations at conferences, several other LKS departments and a principal research radiographer in the NHS have taken up the workshop plans and resources and adapted them to use with staff in their organizations. As these resources are publicly available, other organizations may also have adopted them, but we are unable to capture these data from the website where they are hosted.

Overall, the program was delivered as planned and well received despite smaller participant numbers than hoped. Delivering profession specific training meant that content could be tailored effectively to the participants. Participants not only reported increased knowledge and skills but showed tangible research outputs as a result of being involved in the program. We recommend this style of delivery to other organizations who wish to deliver a research engagement program and welcome individuals or teams to use the resources we developed [7].

## SUPPORTING AGENCIES

Chartered Institute of Library and Information Professionals (CILIP): The Library and Information Association Information Literacy Group (ILG); Lancashire Teaching Hospitals NHS Foundation Trust; and University of Central Lancashire.

## SUPPLEMENTAL FILE

**Appendix:** Full list of workshops offered to potential participants at initial planning stage of program
