# Supplementary material for: Facilitating research amongst radiographers through information literacy workshops
Source: J Med Libr Assoc. 2021 Jan 1;109(1):112–9. doi: 10.5195/jmla.2021.842 (PMC7772987; doi:10.5195/jmla.2021.842)
Supplement: Supplementary file 1 — Appendix: Full list of workshops offered to potential participants at initial planning stage of program [file jmla-109-1-112-s01.pdf]

JMLA Journal of the Medical Library Association

SC
Supplemental content to
J Med Libr Assoc. Jan;109(1):dx.doi.org/10.5195/jmla.2021.842
www.jmla.mlanet.org
© Hurt, Mcloughlin 2021

Facilitating research amongst radiographers through information literacy workshops

Emily Hurt; Alison Mcloughlin

APPENDIX

Full list of workshops offered to potential participants at initial planning stage of program

Advanced Searching in Bibliographic Databases
Creating a Poster
Evaluating Information on the Web
Finding and Applying for Sources of Funding
Introduction to Critical Appraisal
Introduction to Interpreting Statistics
Introduction to Searching Bibliographic Databases
Managing Information and Using Reference Management Software
Overcoming Barriers to Starting Research
An Overview of E-Journals and Databases
Planning Your Research Project
Presentation Skills
Time Management
What to Do with Your Research Idea
Writing an Abstract
Writing for Publication
